# Assessment of Carbon Density in Natural Mountain Forest Ecosystems at Northwest China

**DOI:** 10.3390/ijerph18042098

**Published:** 2021-02-21

**Authors:** Li Dai, Yufang Zhang, Lei Wang, Shuanli Zheng, Wenqiang Xu

**Affiliations:** 1 School of Geographic and Environmental Sciences, Guizhou Normal University, Guiyang 550001, China; daili_helen@163.com; 2 Xinjiang Institute of Ecology and Geography, Chinese Academy of Sciences (CAS), Urumqi 830011, China; 3 Xinjiang Academy of Forestry Sciences, Urumqi 830011, China; wanglei4970@sina.com; 4 College of Resources and Environment Science, Xinjiang University, Urumqi 830046, China; zsl900615@163.com; 5 State Key Laboratory of Desert and Oasis Ecology, Xinjiang Institute of Ecology and Geography, Chinese Academy of Sciences (CAS), Urumqi 830011, China

**Keywords:** biomass, carbon density, field measurements, spatial distribution

## Abstract

The natural mountain forests in northwest China are recognized as a substantial carbon pool and play an important role in local fragile ecosystems. This study used inventory data and detailed field measurements covering different forest age groups (young, middle-aged, near-mature, mature, old-growth forest), structure of forest (tree, herb, litter and soil layer) and trees (leaves, branches, trunks and root) to estimate biomass, carbon content ratio, carbon density and carbon storage in Altai forest ecosystems. The results showed that the average biomass of the Altai Mountains forest ecosystems was 126.67 t·hm^−2^, and the descending order of the value was tree layer (120.84 t·hm^−2^) > herb layer (4.22 t·hm^−2^) > litter layer (1.61 t·hm^−2^). Among the tree parts, trunks, roots, leaves and branches accounted for 50%, 22%, 16% and 12% of the total tree biomass, respectively. The average carbon content ratio was 0.49 (range: 0.41–0.52). The average carbon density of forest ecosystems was 205.72 t·hm^−2^, and the carbon storage of the forest ecosystems was 131.35 Tg (standard deviation: 31.01) inside study area. Soil had the highest carbon storage (65.98%), followed by tree (32.81%), herb (0.78%) and litter (0.43%) layers. Forest age has significant effect on biomass, carbon content ratio, carbon density and carbon storage. The carbon density of forest ecosystems in study area was spatially distributed higher in the south and lower in north, which is influenced by climate, topography, soil types and dominant tree species.

## 1. Introduction

Since the 20th century, the greenhouse effect caused by continuously increased atmospheric CO_2_ concentrations has becoming a serious problem worldwide [1]. Terrestrial carbon sequestration has a potential role in reducing atmospheric CO_2_, which could effectively slow down the speed of greenhouse effect [2]. Forest carbon storage, especially the mid- and high-latitude forests in the Northern Hemisphere, is the largest terrestrial carbon pool on Earth [3,4]. Forests play an important role in maintaining the global carbon balance, mitigating the global climate change effect, and reducing some public health issues [5]. Evaluating the carbon storage, carbon density and spatial distribution of carbon in forest ecosystems could provide a scientific basis for management and sustainable development of forest and environment.

Although carbon storage studies of forest ecosystems have been carried out all over the world, the estimated carbon density values vary due to the wide diversity of vegetation types, forest age, tree structure, climate, regional characteristics, research scales, data sources and applied methodologies [6,7,8,9,10]. Therefore, detailed analysis and field measurements are indispensable for forest carbon estimation to mitigate the methods-induced errors (e.g., allometric equations, atmospheric inverse model, terrestrial ecosystem models and forest inventories) and to help further understand the drivers of variation [3,5,6,11,12].

To further understand forest carbon storage, the more precise carbon sequestration studies focusing on various forest ecosystems in different regions are needed. Northwest China is located in the hinterland of the Eurasian continent. It has a typical inland desert environment and desert irrigation-based economy, which causes simple and vulnerable ecosystem structure with low biomass in natural ecosystems. Local forests play an essential role as ecological barriers for improving the environment, maintaining ecological balance, and ensuring the sustainable development of oasis [13]. The natural mountainous forest in the Altai Mountains has rich vegetation resources and high biodiversity in northwest China and regulates local rivers and floods [14]. Most forest studies of the Altai Mountains focus on tree rings and climate change effects [15,16,17]; the carbon storage of local forest and its distribution pattern are not fully analyzed.

Therefore, the aim of this study is to carry out comprehensive and in-depth carbon research of the forest ecosystems in Altai Mountains, which has great significance on forest resources conservation and maintaining the balance of the “Mountain–Oasis–Desert” ecosystem in arid central Asia. Our research was based on the field survey and the inventory data, and analyzed carbon density and carbon storage of the forest ecosystems in the Altai Mountains. The following hypotheses were tested:

(1) The biomass, carbon content ratio, carbon density and carbon storage in different parts of forest ecosystems, such as trunk, branch, leaf, herb, etc., have no obvious difference in age groups (H_0_), or they are highly related to age groups (H_1_) of the natural mountain forest inside study area.
(2) The variation of tree species (composition and DBH pattern) is not related to forest age groups and soil carbon (H_0_), or there is a relation (H_1_) in the natural mountain forest of arid central Asia.

By testing the hypotheses, the patterns of biomass, carbon content ratio, carbon density, carbon storage and tree species composition were analyzed, and results should be beneficial to policy-makers and local residents for understanding organic carbon pool mechanism and designing sustainable development strategies in arid regions.

## 2. Materials and Methods

### 2.1. Study Area

**Figure 1:**
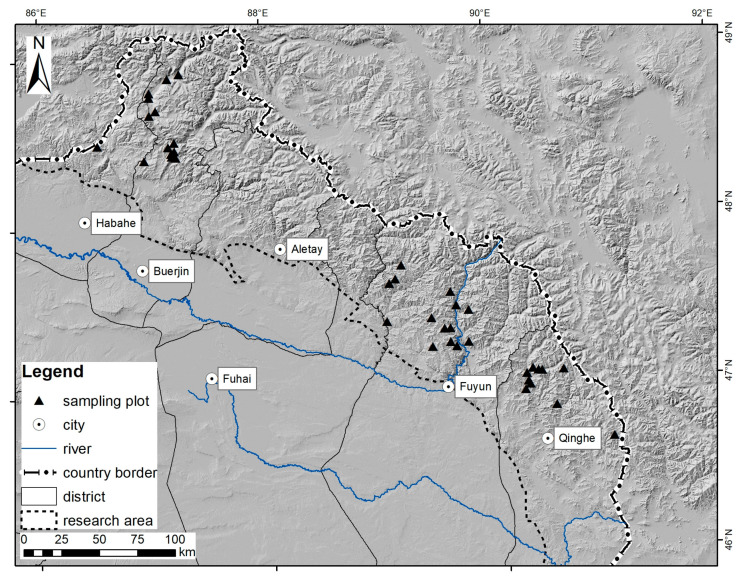
Location of study area and sampling plots in the Altai Mountains.

The Altai Mountains are a mountain range located in Central and East Asia, where Russia, China, Mongolia, and Kazakhstan meet and stretch for approximately 2000 km in length. The mountains are high and wide at the northwest end, and gradually become narrow and lower and merges into the high plateau of the Gobi Desert at the southeast end. The study area is the southern slope of middle Altai Mountains inside Xinjiang province of China, which extends more than 500 km in length (Figure 1).

The local climate type is temperate continental climate, which is warm and rainy in summer, cold and dry in winter. Because of the airflow from the Atlantic and Arctic Ocean, a rainy zone was formed at the mid-mountain belt (1100–2300 m a.s.l.), where the forest has developed. Within forest zone, the annual mean temperature is −4.0–2.0 °C, and the annual precipitation is 400–600 mm, which increases with the altitude rises, and decreases from northwest to southeast [18].

The local mountain coniferous forest is the south end of the Siberian taiga, which represents the boreal coniferous forest wedging into the prairies [19]. The dominate species of coniferous forest is *Larix sibirica*, which distributed on the shady and semi-shady slopes [19]. It also mixes with *Abies sibirca* at the humid northern slopes, and with a few *Picea obovate* in river valleys at the lower altitude. The deciduous forest is mainly dominated by *Betula pendula*, *Populus tremula*, etc. There were only a few disturbances inside the forest of the study area: at the lower limit of the forest, a few loggings and small tree planting in 1950s [19].

The brown coniferous forest soil and gray forest soil are the main soil types beneath the forest [19]. Soil parent materials are mostly lithic matters caused by slope colluviation effect, and a small amount of residual carbonate deposited on rocky parent materials [19].

### 2.2. Data Collection and Analysis

#### 2.2.1. Field Sampling and Measurements

The data used in this study were collected by field measurements and from dataset of Xinjiang forest resources survey. The survey of Xinjiang forest resources was carried out in 2006, and part of the Chinese 7th National Forest Resources Inventory [20]. Considering the distribution and variability of local climate, topography and vegetation, approximately 210 grids (10 × 10 km per grid) were set covering most forest area (arbor forest) in Xinjiang province. Among them, 70 grids were inside research site of this study. Forest area, age and stand volume were investigated in each grid.

Considering accessibility, watershed and forest distribution, etc., 35 grids were selected to set sampling plots for detailed measurements in the year of 2011 [21]. In each grid, one sampling plot (800 m^2^, 28.3 m × 28.3 m) was placed (Figure 1) to investigate status of tree, herb, litter and soil layer, respectively. Considering forest age, area and stand volume, these 35 sampling plots were set to make sure covering different age groups of natural forest (Table 1) [22]. The basic information of these sampling plots is in Table 2.

Inside each sampling plot, tree, herb, litter and soil layer were investigated with different methods:

- Soil: one soil profiles were randomly selected in each sampling plot. At each profile, three soil samples were collected at each depth (0–10, 10–20, 20–30, 30–50 and 50–100 cm). If the soil depth of some locations was less than 100 cm, the soil samples were taken until the soil parent materials was reached. The soil samples were air-dried, crushed and passed through a sieve, and then the soil organic matter (OM) was determined by potassium dichromate external heating method.
- Trees: diameter at breast height (DBH) (at 1.3 m above ground) and tree height were measured for all the trees which DBH was larger than or equal to five centimeters (DBH ≥ 5 cm) [23]. Therefore, 2348 trees were measured in 35 sampling plots.

Within these 35 plots, nine plots were selected covering different forest age groups. Twenty-seven trees were randomly selected in these nine plots (three trees in each plot), parts of leaves, trunks, branches and roots of these 27 trees were collected as samples. These samples’ fresh weights were measured at field, and then brought back to laboratory for dry weight and other analysis. Considering there are only a few dead trees in the sampling plots, tree parts of dead trees were collected and measured if they were found in the field. These samples were included in the tree layers in this study.

- Herb and litter layers: three small quadrats (1 m × 1 m) were randomly selected in each sampling plot (800 m^2^). Inside 105 quadrats, all the above-ground part and underground roots of grass, and all the litters were collected. The fresh weight was measured at field, and dry weight was measured in the lab.

All the tree (leaves, branches, trunks and roots), herb and litter samples were air-dried, crushed and passed through a 0.15-mm sieve, and their organic carbon was determined by using potassium dichromate oxidation methods.

#### 2.2.2. Biomass Calculation

Biomass of herb and litter layers were directly calculated from field sampling and measurements in laboratory. Tree biomass was estimated by an allometric equation [24]:

*W* = *a*(*D*^2^*H*)^*b*^ (1)

where *W*, *D* and *H* represent the biomass per tree, DBH and tree height, respectively; *a* and *b* are parameters.

For each part of tree (leaves, branches, trunks and roots), biomass was calculated separately according to tree species [25] (Table 3). Then, tree biomass was estimated by adding them (biomass of different tree organs) together.

#### 2.2.3. Forest Carbon Density and Carbon Storage

Carbon density (CD) of tree, herb and litter layers were calculated by multiplying biomass and carbon content ratio (CCR), and then adding them (CD of tree, herb and litter layers) together.

The soil CD (0–100 cm) was the sum of each layer’s CD. The soil organic CD (*S_i_*_)_ of each layer was calculated as [26]:

*S_i_* = *C_i_* × *D_i_* × *E_i_* × (1 − *G_i_*) (2)

where *C_i_* represents the CCR, *D_i_* is soil bulk density, *E_i_* is soil thickness, *G_i_* is the volume percentage of the gravels with the diameter of more than 2 mm.

The CD of forest ecosystems was calculated by adding tree, herb, litter and soil CD together. Together with these plots’ information, we estimated the carbon density values of all the survey plots in Haba River, Buerjin, Fuyun, and Qinghe forest districts, respectively, from northwest to southeast of Altai Mountains.

The carbon storage of forest ecosystems was estimated by multiplying CD of the forest ecosystems and the area of forest. Forest area was obtained from Xinjiang forest resources survey.

The statistical analysis and correlation tests were carried out in Excel and SPSS. The effects of different forest age groups on biomass and carbon storage were analyzed using the one-way analysis of variance (ANOVA) and least significant difference (LSD) test.

## 3. Results

### 3.1. Carbon Content Ratio and Biomass

The average CCR of the Altai Mountains forest ecosystems was 0.49 (range: 0.41–0.52), which was significantly influenced by forest age (Table 4). CCR of tree and litter layer were similar, and much higher than that of herb. The tree organ with highest CCR was trunk, followed by leaf and branch, and root had the lowest CCR. The CCR were significantly different among forest age groups: both mature and old-growth forest had the highest CCR for all vegetation layers; young forest had relatively higher CCR for herb and litter layers.

The average biomass of the Altai Mountains forest ecosystems was 126.67 t·hm^−2^ (Table 5). The highest biomass was in trees (accounting for 95.14% of total biomass), followed by grass; the litter layer had the lowest biomass. Among the tree organs, biomass of trunk was the highest (>50%), followed by root (22%) and leaves (16%), and branch shared the smallest proportion (12%). Forest age had great impact on the trunk biomass (*p* < 0.05), and the difference between young and mature/old-growth forest was significant. Biomass of the entire vegetation layer increased with forest age: biomass of per unit area in mature and old-growth forest was the highest, while young forest was the lowest. Biomass of tree and litter were positively related to forest age, while herb biomass peaked in young forest (Table 5; Figure S1).

### 3.2. Carbon Density and Carbon Storage

Inside study area, the average CD of forest ecosystems was 205.70 t·hm^−2^, and the average soil CD was 141.29 t·hm^−2^ (more than twice of the rest CD) (Table 6). The average CD of tree layer was much higher than that of herb and litter layers, and accounted for more than 95% of the total vegetation CD. Among the tree organs, trunk had the highest average CD (51%), which branch had the lowest (11%).

The vegetation CD was positively related to forest age, while the soil CD peaked in middle-aged forest (Table 6; Figure S2). Forest age had substantial impact on the CD of root and trunk (*p* < 0.05): for trunk, the difference between young and mature/old-growth forest was significant; for root, differences between young and mature/old-growth forest, and the differences between near-mature forest and old-growth forest were significant. The CD of trunk and root were increased with increasing forest age, while CD of leaf and branch were peaked in young and middle-age forest (Table 6; Figure S3). The high soil CD and its standard deviation in the middle-aged forest might be induced by the unproper selection and the relatively fewer number (three plots) of sampling plots at this age group. The similar average soil CD from near-mature to old-growth forest could be the result of the high standard deviation of these values.

The carbon storage of the Altai Mountains forest ecosystems was 131.35 Tg (standard deviation: 31.01; Table 6). Soil carbon storage was the highest (86.67 Tg), followed by tree layer (43.09 Tg) and herb layer (1.03 Tg), and litter was the lowest (0.56 Tg). The carbon storage of soil and trees (accounted for 65.98% and 32.81% of the total carbon storage) were the major carbon pools of Altai Mountains forest ecosystems. Forest age was closely related to carbon storage, and mature and old-growth forest had the highest carbon storage.

### 3.3. Spatial Distribution of Carbon Density

Using the sampling plots information, forest carbon density was calculated in Haba River-Buerjin, Fuyun, and Qinghe forest districts, respectively, from northwest to southeast of Altai Mountains (Table 7). Habahe was combined with Buerjin since there was only one plot in Habahe region. The carbon density distribution of Altai Mountains forest ecosystems was higher in south and lower in north: Qinghe had the highest carbon density, followed by Buerjin, and Habahe-Buerjin.

## 4. Discussion

### 4.1. The Factors Influencing Biomass and Carbon Density Estimations

The accuracy of biomass calculation is the key factor for carbon storage estimation of forest ecosystems. However, the differences of biomass estimation were induced by calculation methodology and research sites: Liu et al. [27] assessed the tree layer biomass of northwest China as 104.31 t·hm^−2^ using remote sensing methods; Wang et al. [28] calculated larch biomass of Greater Khingan as 48.64–137.68 t·hm^−2^ based on the biomass equation. These values were different from the biomass calculated in this study. The main cause of this variation is the estimation of old-growth forest biomass, while the values for other forest age groups are similar. Within the Altai mountain forest, the biomass of the vegetation layer positively related to forest age, i.e., the young forest has the lowest biomass, and the difference between young and mature/old-growth forest was significant [29].

To calculate CD, the value of 0.50 and 0.45 are usually used as CCR for trees and understory vegetation, respectively [30]. However, in this study, the average CCR is 0.51 for tree and litter layers, and is 0.41 for herb layer. The CCR also varies among different tree organs and forests of different age groups in Altai Mountains. Therefore, the detailed measurements of CCR in different forest types could improve the accuracy of forest carbon storage calculation [5,12]. Meanwhile, the samples of dead trees were included in the tree layer in the field work of this study since there were only a few dead trees found in the sampling plot. This should be improved by separating dead tree samples in the future studies of this area.

The values of average forest CD vary among studies. the continuous biomass expansion factor method was used to build linear and hyperbolic models of biomass-stand volume and calculated the average CD of vegetation as 41.00 t·hm^−2^ or 41.32 t·hm^−2^ [3,28,31]. Cheng et al. [32] used biomass standard data and the measured CCR to estimate the mean CD of *Larix kaempferit* vegetation, and the result was 33.04 t·hm^−2^. Zhou et al. [33] calculated the average carbon density of larch and the result was 60.20 t·hm^−2^. Some of these forest carbon density values are different from the result of this study (64.49 t·hm^−2^) at the range of approximately 20%, some are similar. The variation may be caused by different methods and vegetation types.

### 4.2. The Causes of Forest Carbon Density Distribution

The carbon density distribution of forest ecosystems is higher in south and lower in north inside study area. This spatial pattern is determined by many factors, such as climate, topography, vegetation, soil types, etc. [34,35,36].

The Altai Mountains lie in a northwest–southeast direction, forming a horn shape with the high and wide end to the northwest, and a narrow and lower end to the southeast merging into the Gobi Desert. Along the Irtysh River valley at the Altai Mountains piedmont, the cold and wet airflows of Atlantic and Arctic oceans from the northwest are the only moist airflow sources for the regions. The mountains in the northwest intercept most of the airflows, and therefore the climate is humid and warm comparing to other parts of the study area. The local forest coverage is relatively low, but with high soil organic carbon. In the middle region of the Altai Mountains, forest is widely distributed in Aletay, Fuhai and Fuyun, where is cooler and less humid than the northwest. At the southeast, due to the decreasing altitude and the effects of Siberia Mongolia high pressure dry anticyclone, the climate is cold and dry. Although the forest area decreases in Qinhe region at southeast, the local carbon density of forest ecosystems is relatively high because of the high coverage ratio of mature and old-growth forest [6].

Inside study area, the brown coniferous forest soil and gray forest soil are main soil types; both of them have high soil organic carbon. Gray forest soil is the most widely distributed forest soil in Altai mountains, which is suitable for growing *Larix sibirica* [19]. The forests develop on this soil type are mainly mature and old-growth forest. At the southeast of the study area, the forest ecosystems have highest carbon density in Qinhe region because of the combination of forest age groups (mature and old-growth forest) and soil types (gray forest soil).

The change of dominant species also coincides with the spatial pattern of vegetation carbon density inside study area [12]. From Buerjin in northwest to Qinghe in southeast, the proportion of dominant species, *Larix sibirica* and *Picea obovate*, increase from 72.1% to 100.0% (the forest in Habahe is eliminated for detailed analysis of dominant species since there is only one sampling plot in this area). The number of *Larix sibirica* increases greatly, while the trend of *Picea obovate* is minor (Table 2).

*Larix sibirica* has strong adaptability, as it is cold and frost resistant, with barren tolerance and accommodated to rocky soil. In Fuyun and Qinghe in the southeast part of study area, the weather is cold and dry, and the rocky soil is low in organic matter, which leads to a large proportion of *Larix sibirica*. However, *Larix sibirica* is photophilous, and its natural regeneration ability is poor in mixed forests of larch and spruce with high canopy coverage. It can be easily replaced by *Picea obovata* due to disturbances caused by human activities (logging) and fires [37]. The forest of sampling plots in Qinghe is mixed forest of only *Larix sibirica* and *Picea obovata*. In addition, the forest type is mature and old-growth forest, which leads to the highest carbon density inside study area.

### 4.3. Forest Carbon Storage and Forest Structure

The studies on carbon storage and forest age of Altai Mountains forest ecosystems showed that carbon storage was significantly correlated with forest age, meaning that the carbon storage increases with forest age increases, which is consistent with the common pattern in China [32]. The Altai Mountains forests mostly consisted of mature and old-growth forest, which accounted for 32% and 30% of local forest area, respectively. The carbon storage of these two types were 32% and 29% of the total ecosystems carbon storage, which has the similar pattern with their area ratio (Table 6). With the increase amount of old-growth forest, the carbon storage of the entire region will also increase. Even considering the death of old-growth trees, the forest carbon storage will keep the high concentration because of the long-term decomposition period (decades-to-century) and carbon conversion from vegetation to soil [38,39].

**Figure 2:**
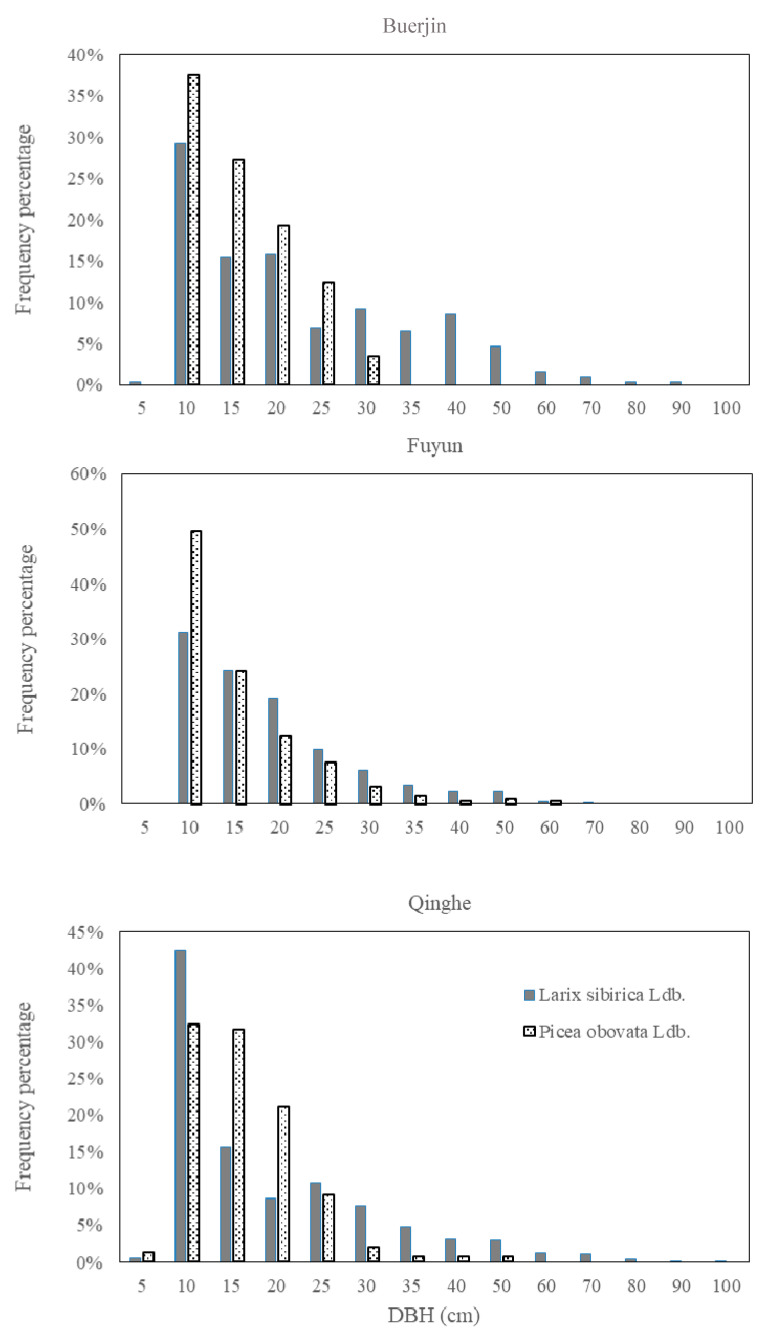
The DBH (the trunk diameter at 1.3 m from the base) of dominant species in different forest districts of the Altai Mountains.

Additionally, the DBH of dominant species in different region inside study area shows that: the number of *Larix sibirica* increase from northwest to southeast (Table 2), and the DBH of *Larix sibirica* reaches peak (31–42%) around 10 cm (Figure 2). This implies a potential increase of carbon storage of the Altai Mountains in the future [7,39].

## 5. Conclusions

Mountain forests in arid northwest China function as a substantial carbon pool, and have great significance for forest resource conservation and in maintaining the balance of the local “mountain–oasis–desert” ecosystem. In this study, we used detailed field measurements covering different forest age groups, structure of forest (tree, herb, litter and soil layer) and trees (leaves, branches, trunks and root) to calculate biomass, carbon density and carbon storage in the Altai forest ecosystems. The major conclusions can be summarized as follows:

(1) The average biomass of the Altai Mountains natural forest ecosystems is 126.67 t·hm^−2^. Trees have the highest biomass (120.84 t·hm^−2^), followed by herb (4.22 t·hm^−2^) and litter layer (1.61 t·hm^−2^). Among the tree parts, trunks, roots, leaves and branches accounted for 50%, 22%, 16% and 12% of the total tree biomass, respectively.
(2) The average carbon content ratio is 0.49, the average carbon density is 205.72 t·hm^−2^, and the carbon storage is 131.35 Tg in Altai Mountains forest ecosystems. Soil and tree layers are the main local carbon pools.
(3) Forest age has significant effect on biomass, carbon content ratio, carbon density and carbon storage. For parts of tree and forest ecosystems, forest age effect for carbon content ratio was the most obvious, while the biomass of trunk and carbon density of trunk and root were significantly correlated with forest age.
(4) The change of dominant species coincides with the spatial pattern of vegetation carbon density inside study area, which implies a potential increase of local carbon storage in the future.

## Figures and Tables

**Table 1:** The information of sampling plots according to the forest age in Altai Mountains natural forests.

Age Group	Age (a)	No. of Tree Samples	Forest Area Statistics		DBH (cm)		Tree Height (m)	
			Area (×10^4^ hm^2^)	Proportion (%)	Range	Mean	Range	Mean
Young forest	≤40	144	1.42	2.27	9.20–12.50	10.85	8.10–9.10	8.6
Middle-aged forest	41–80	316	9.99	15.93	10.06–15.06	12.72	8.43–15.01	11.10
Near-mature forest	81–100	568	12.25	19.54	12.08–18.65	15.55	9.82–13.40	11.87
Mature forest	101–140	427	20.09	32.04	14.00–21.01	17.83	11.42–14.66	12.89
Old-growth forest	>140	893	18.94	30.22	15.48–37.55	24.32	11.15–18.30	14.67

**Table 2:** The distribution pattern of dominant species in sampling plots of the Altai Mountains.

Region	No. of Sampling Plot	Average Number of Trees in the Plots	Dominant Species		Mean of the DBH (cm)	Mean of the Tree Height (m)	Vegetation Carbon Density (t·hm^−2^)
			Type	Ratio to the Trees in Plots (%)			
Habahe	1	52	*Larix sibirica Ldb.*	9.6	50.9	23.5	76.86
			*Picea obovata Ldb.*	88.5	11.3	11.1	
Buerjin	11	49	*Larix sibirica Ldb.*	56.0	20.5	14.3	66.03
			*Picea obovata Ldb.*	16.1	13.1	10.4	
Fuyun	13	83	*Larix sibirica Ldb.*	68.5	16.1	12.6	66.11
			*Picea obovata Ldb.*	19.7	12.5	10.1	
Qinghe	10	68	*Larix sibirica Ldb.*	79.3	17.2	12.7	78.76
			*Picea obovata Ldb.*	20.7	13.5	10.1	
mean		63			19.4	13.1	71.94

**Table 3:** The biomass equations for dominant species.

Dominant Species	Biomass Equation	R^2^	DBH Range (cm)
*Larix sibirica*	*W_S_* = *0.099496(D*2*H)*0.78653	0.990	1.2~37.0
	*W_B_* = *0.098620(D*2*H)*0.598367	0.990	
	*W_L_* = *0.294136(D*2*H)*0.357506	0.990	
	*W_R_* = *0.00698(D*2*H)*0.9724	0.998	
*Picea obovata* *Abies sibirca*	*W_S_* = *0.1283(D*^2^*H)*^0.7534^	0.913	4.3~128.4
	*W_B_* = *0.093(D*^2^*H)*^0.6732^	0.913	
	*W_L_* = *0.7753(D*^2^*H)*^0.5903^	0.913	
	*W_R_* = *0.1002(D*^2^*H)*^0.6674^	0.913	
*Betula pendula* *Populus tremula*	*W_S_* = *0.6039(D*^2^*H)*^0.5325^	0.959	1.5~69.2
	*W_B_* = *1.016(D*^2^*H)*^0.3922^	0.957	
	*W_L_* = *0.6989(D*^2^*H)*^0.2475^	0.960	
	*W_R_* = *0.8207(D*^2^*H)*^0.3878^	0.956	

Note: *D*, DBH (cm); *H*, tree height (m); *W_S_*, tree trunk biomass (Kg); *W_B_*, tree branch biomass (Kg); *W_L_*, tree leaf biomass (Kg); *W_R_*, tree root biomass (Kg).

**Table 4:** The carbon content ratios (CCR) in different forest age groups.

Age Group	Tree				Herb	Litter
	Trunk	Branch	Leaf	Root		
Young forest	0.52 (0.02) ^A,B^	0.50 (0.02) ^B,C^	0.50 (0.02) ^A,B^	0.49 (0.02) ^B^	0.42 (0.04) ^a,b,c^	0.51 (0.01) ^A,B^
Middle-aged forest	0.51 (0.02) ^B^	0.49 (0.01) ^C^	0.50 (0.02) ^B,C^	0.48 (0.02) ^B^	0.39 (0.04) ^c^	0.50 (0.01) ^B^
Near mature forest	0.51 (0.01) ^B^	0.49 (0.01) ^C^	0.50 (0.02) ^B^	0.49 (0.02) ^B^	0.40 (0.03) ^c^	0.50 (0.01) ^B^
Mature forest	0.52 (0.00) ^A^	0.53 (0.01) ^A^	0.51 (0.01) ^A,C^	0.51 (0.01) ^A^	0.42 (0.01) ^b^	0.52 (0.01) ^A^
Old-growth forest	0.53 (0.00) ^A^	0.51 (0.03) ^A,B^	0.52 (0.01) ^A^	0.51 (0.01) ^A^	0.43 (0.01) ^a^	0.52 (0.01) ^A^
Mean	0.52 (0.01)	0.50 (0.02)	0.51 (0.02)	0.50 (0.02)	0.41 (0.03)	0.51 (0.01)
*F* value	4.03 **	7.71 **	4.12 **	12.37 **	3.44 *	5.32 **

The values in the brackets are standard deviation; Letters represent the difference between group: if the letters are the same, there is no difference; if the letters are different, the difference is significant; * *p* ≤ 0.05, the differences among age groups are represented by the lowercase letters; ** *p* ≤ 0.01, the differences among age groups are represented by capital letters.

**Table 5:** The biomass per unit area (t·hm^−2^) in Altai Mountains forests.

Age Group	Tree				Herb	Litter	Total
	Trunk	Branch	Leaf	Root			
Young forest	31.92 (24.24) ^b^	8.78 (4.29)	24.56 (10.68)	14.45 (12.91)	6.34 (5.15)	1.33 (0.87)	87.38 (55.49)
Middle-aged forest	51.46 (17.03) ^a,b^	16.56 (8.50)	23.57 (16.98)	21.83 (9.72)	3.00 (3.99)	1.37 (1.55)	117.79 (40.38)
Near mature forest	62.25 (18.48) ^a,b^	14.46 (5.54)	20.10 (15.85)	24.84 (6.81)	3.88 (4.93)	1.76 (1.11)	127.29 (32.05)
Mature forest	80.86 (26.39) ^a^	15.07 (4.84)	12.08 (10.47)	32.94 (12.13)	4.23 (3.88)	1.77 (0.72)	146.95 (44.78)
Old-growth forest	78.19 (31.56) ^a^	14.93 (7.31)	17.82 (20.43)	37.47 (18.15)	3.63 (2.52)	1.85 (1.01)	153.89 (66.34)
Mean	60.94 (28.51)	13.96 (5.93)	19.63 (15.12)	26.31 (14.19)	4.22 (3.81)	1.61 (0.93)	126.67 (50.49)
*F* value	2.98 *	0.81	0.70	2.56	0.34	0.16	

The values in the brackets are standard deviation; Letters represent the difference between groups: if the letters are the same, there is no difference; if the letters are different, the difference is significant; * *p* ≤ 0.05, the differences among age groups are represented by the lowercase letters.

**Table 6:** The carbon density and carbon storage of Altai Mountains forest ecosystems.

Age Group	Carbon Density (t·hm^−2^)							Carbon Stock (Tg)
	Tree				Herb	Litter	Soil	
	Trunk	Branch	Leaf	Root				
Young forest	16.76 (12.98) ^b^	4.43 (2.29)	12.42 (5.55)	7.24 (6.60) ^c^	2.77 (2.28)	0.67 (0.47)	134.26 (12.98)	2.55 (0.69)
Middle-aged forest	26.16 (8.08) ^a,b^	8.04 (4.05)	11.55 (8.13)	10.45 (4.41) ^a,b,c^	1.28 (1.77)	0.70 (0.81)	171.72 (110.03)	22.96 (6.18)
Near mature forest	31.82 (9.26) ^a,b^	7.08 (2.77)	10.08 (8.12)	11.98 (3.17) ^b^	1.65 (2.17)	0.89 (0.56)	145.92 (53.55)	25.65 (6.39)
Mature forest	42.40 (13.88) ^a^	7.93 (2.56)	6.17 (5.39)	16.91 (6.19) ^a,b^	1.81 (1.65)	0.92 (0.38)	132.86 (56.92)	41.99 (9.56)
Old-growth forest	41.28 (16.53) ^a^	7.68 (3.84)	9.29 (10.57)	19.24 (9.36) ^a^	1.58 (1.11)	0.96 (0.52)	121.67 (35.85)	38.21 (8.19)
Total								131.35 (31.01)
Mean	31.68 (15.00)	7.03 (3.07)	9.90 (7.72)	13.16 (7.36)	1.82 (1.66)	0.82 (0.48)	141.29 (53.90)	
*F* value	3.19 *	0.85	0.64	2.96 *	0.35	0.18	0.68	

The values in the brackets are standard deviation; Letters represent the difference between groups: if the letters are the same, there is no difference; if the letters are different, the difference is significant; * *p* ≤ 0.05, the differences among age groups are represented by the lowercase letters.

**Table 7:** The carbon density distribution of Altai Mountains forest ecosystems (t·hm^−2^).

Region	Lower Limit of Tree Line (m a.s.l.)	Upper Limit of Tree Line (m a.s.l.)	Carbon Density (t·hm^−2^)		
			Vegetation	Soil	Forest Ecosystem
Habahe-Buerjin	1000	2200	66.93 (24.65)	118.79 (33.27)	185.72 (45.50)
Fuyun	1300	2400	66.11 (23.94)	127.87 (61.57)	193.98 (66.16)
Qinghe	1600	2500	78.76 (32.23)	156.68 (60.32)	235.44 (54.38)

The values in the brackets are standard deviation.

## Supplementary Materials

The following are available online at https://www.mdpi.com/1660-4601/18/4/2098/s1, Figure S1: The biomass in different forest age groups, Figure S2: The carbon density of forest layers in different forest age groups, Figure S3: The carbon density of tress parts in different forest age groups.

## References

[1] Griggs D.J., Noguer M. (2002). Climate change 2001: The scientific basis. Contribution of Working Group I to the Third Assessment Report of the Intergovernmental Panel on Climate Change. Weather.

[2] Guang Q., Qi G., Wang Q., Wang X., Yu D., Zhou L., Zhou W., Peng S., Dai L. (2013). Soil organic carbon storage in different aged Larix gmelinii plantations in Great Xing’an Mountains of Northeast China. Chin. J. Appl. Ecol..

[3] Fang J., Chen A., Peng C., Zhao S., Ci L. (2001). Changes in Forest Biomass Carbon Storage in China between 1949 and 1998. Science.

[4] Chen Y., Luo G., Ye H., Wang Y., Huang X., Zhang Q., Cai P. (2015). Sources and Sinks of Carbon Caused by Forest Land Use Change from 1975 to 2005 in Central Asia. J. Nat. Resour..

[5] Nowak D., Crane D. (2002). Carbon storage and sequestration by urban trees in the USA. Environ. Pollut..

[6] McGarvey J.C., Thompson J.R., Epstein H., Shugart H. (2015). Carbon storage in old-growth forests of the Mid-Atlantic: Toward better understanding the eastern forest carbon sink. Ecology.

[7] Wang W., He H., Thompson F., Fraser J., Dijak W. (2017). Changes in forest biomass and tree species distribution under climate change in the northeastern United States. Landsc. Ecol..

[8] Zhang C., Ju W., Chen J., Zan M., Li D., Zhou Y., Wang X. (2013). China’s forest biomass carbon sink based on seven inventories from 1973 to 2008. Clim. Chang..

[9] Fang J., Chen A. (2001). Dynamic forest biomass carbon pools in China and their significance. Acta Bot. Sin..

[10] Liu W., Wang X., Lu F., Ouyang Z. (2015). Regional and global estimates of carbon stocks and carbon sequestration capacity in forest ecosystems. Chin. J. Appl. Ecol..

[11] Lambert M.-C., Ung C., Raulier F. (2005). Canadian national tree aboveground biomass equations. Can. J. For. Res. Rev. Can. De Rech. For. Can. J For. Res..

[12] Cienciala E., Černý M., Tatarinov F., Apltauer J., Exnerová Z. (2006). Biomass functions applicable to Scots pine. Trees.

[13] Zhao B. (2009). Development status and strategies of forest ecosystem in Xinjiang. For. Xinjiang.

[14] Liang Y., Liu X., Habai Y., Xu G. (2006). The analysis of Forest combustible type in Altai. Forest Fire Prev..

[15] Ni J. (2004). Forest productivity of the Altay and Tianshan Mountains in the dryland, northwestern China. For. Ecol. Manag..

[16] Zhou G., Wang Y., Jiang Y., Yang Z. (2002). Estimating biomass and net primary production from forest inventory data: A case study of China’s Larix forests. For. Ecol. Manag..

[17] Zhang R., Shang H., Yuan Y., Wei W., Zhang T., Chen F., Yu S., Fan Z., Qin L. (2015). Summer precipitation variation in the southern slope of the Altay Mountains recoreded by tree-ring δ^13^ C. J. Desert Res..

[18] Nuerlan H. (2001). Hydrological features of rivers in Altai prefecture. Hydrology.

[19] The Forest Department of Xinjiang Uygur Autonomous Region (1995). Forest Soil of Xinjiang Mountain Region.

[20] Chinese Ministry of Forestry (2009). Forest Resource Report of China—The 7th National Forest Resources Inventory.

[21] IPCC (2006). IPCC Guidelines for National Greenhouse Gas Inventories. http://www.Ipcc-nggip.iges.or.jp/public/2006gl/index.html.

[22] Xu B., Guo Z., Piao S., Fang J. (2010). Biomass carbon stocks in China’s forests between 2000 and 2050: A prediction based on forest biomass-age relationships. Sci. China Life Sci..

[23] Forest Resources Management Division of the State Forestry Bureau (2013). Main Technical Requirements of the National Forest Resources Continuous Inventory.

[24] Luo Y., Zhang X., Wang X., Zhu J., Hou Z., Zhang Z. (2009). Forest Biomass Estimation Methods and Their Prospects. Sci. Silvae Sin..

[25] Office of Forest Carbon Fixation Project (2014). Biomass Estimation Equation.

[26] Liang Q., Yu X., Pang Z., Wang C., Lv X. (2010). Study on soil organic carbon density of different forest types. Ecol. Environ. Sci..

[27] Liu S., Zhou T., Shu Y., Dai M., Wei L., Zhang X. (2012). The estimating of the spatial distribution of forest biomass in China based on remote sensing and downscaling techniques. Acta Ecol. Sin..

[28] Wang X., Chang Y., Chen H., Hu Y., Feng Y., WU W., Jiao L. (2014). Biomass allocation characteristics of the main forest ecosystems in the Great Xing’an Mountains, Heilongjiang Province. Chin. J. Ecol..

[29] Taylor A., Wang J. (2007). Carbon storage in a chronosequence of red spruce (Picea rubens) forests in central Nova Scotia, Canada. Can. J. For. Res..

[30] Wang X., Feng Z., Ouyang Z. (2001). Vegetation carbon storage and density of forest ecosystems in China. Chin. J. Appl. Ecol..

[31] Zhao M., Zhou G. (2004). Carbon Storage of Forest Vegetation and its Relationship with Climatic Factors. Sci. Geogr. Sin..

[32] Cheng T., Feng J., Ma Q., Wang Y., Kang F., Feng Z., Zhang Y., Deng X. (2008). Carbon pool and allocation of forest vegetations in Xiaolong Mountains, Gansu Province. Acta Ecol. Sin..

[33] Zhou Y., Yu Z., Zhao S. (2000). Carbon storage and budget of major Chinese forest types. Acta Phytoecol. Sin..

[34] Tang X., Zhao X., Bai Y., Tang Z., Wang W., Zhao Y., Wan H., Xie Z., Shi X., Wu B. (2018). Carbon pools in China’s terrestrial ecosystems: New estimates based on an intensive field survey. Proc. Natl. Acad. Sci. USA.

[35] Usol’tsev V., Shobairi S.O.R., Chasovskikh V. (2018). Geographic gradients of forest biomass of two needled pines on the territory of Eurasia. Ecol. Quest..

[36] Gu F., Pang R., Zhang Y., Huang M., Li J., Hao W., Mei X. (2014). Temporal-spatial Variations of Soil Organic Carbon and Their Responses to Climate Change in Alpine Area of Southwest China during 1954–2010. J. Nat. Resour..

[37] Zhao Y. (1983). Preliminary study on natural succession law of Larix sibirica forest. Xinjiang Agric. Sci..

[38] Luyssaert S., Schulze E.D., Börner A., Knohl A., Hessenmöller D., Law B., Ciais P., Grace J. (2008). Old-growth forests as global carbon sinks. Nature.

[39] Zhou G., Liu S., Li Z.A., Zhang D., Tang X., Zhou C., Yan J., Mo J. (2007). Old-Growth Forests Can Accumulate Carbon in Soils. Science.

