# Supplementary material for: Assessment of Carbon Density in Natural Mountain Forest Ecosystems at Northwest China
Source: Int J Environ Res Public Health. 2021 Feb 21;18(4):2098. doi: 10.3390/ijerph18042098 (PMC7926412; doi:10.3390/ijerph18042098)
Supplement: Supplementary file 1 [file ijerph-18-02098-s001.pdf]

Supplementary Materials

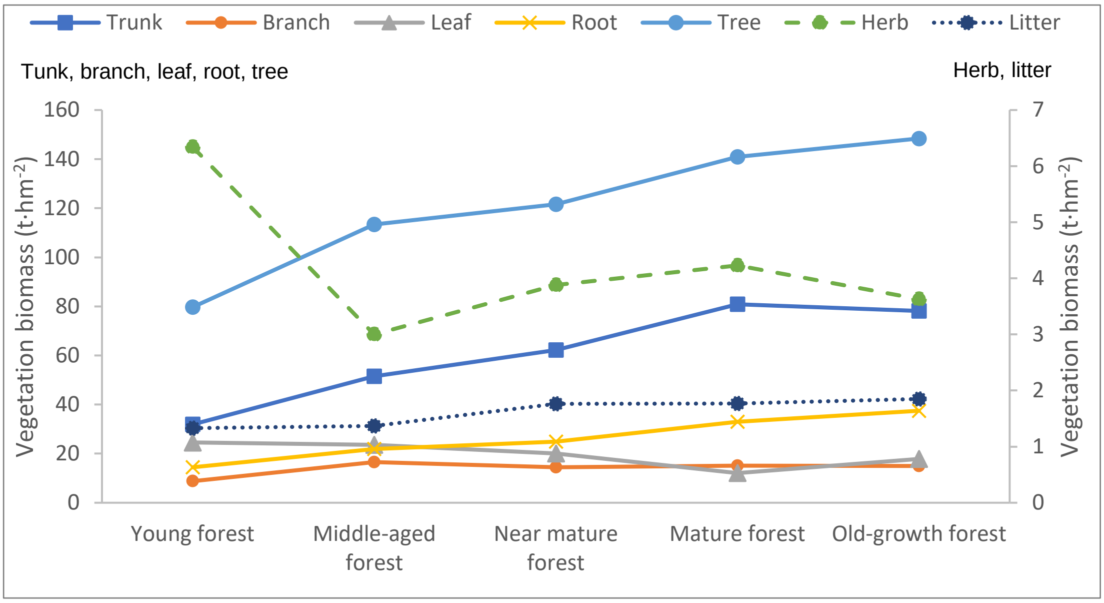

Figure S1. The biomass in different forest age groups.

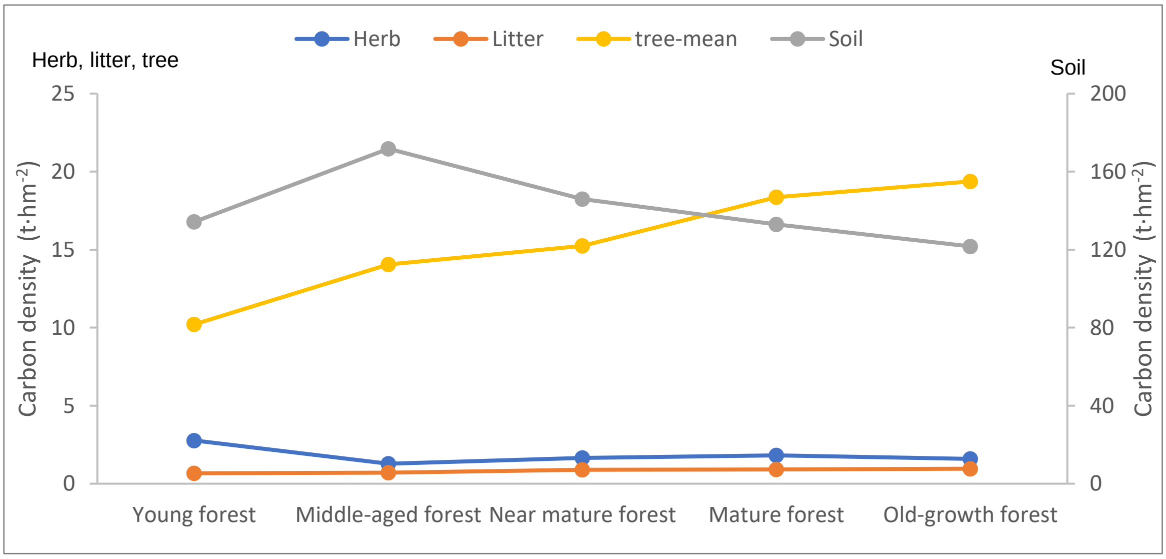

Figure S2. The carbon density of forest layers in different forest age groups.

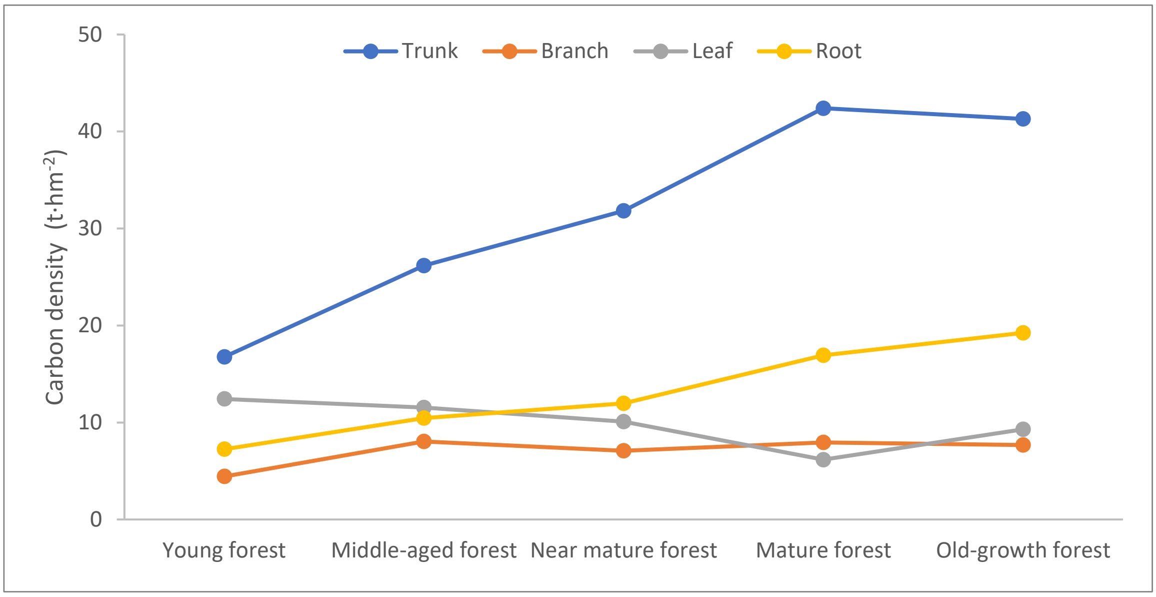

Figure S3. The carbon density of tress parts in different forest age groups.
